# Subcellular Localization of Hexokinases I and II Directs the Metabolic Fate of Glucose

**DOI:** 10.1371/journal.pone.0017674

**Published:** 2011-03-09

**Authors:** Scott John, James N. Weiss, Bernard Ribalet

**Affiliations:** 1 UCLA Cardiovascular Research Laboratory, Department of Physiology, David Geffen School of Medicine at University of California Los Angeles, Los Angeles, California, United States of America; 2 UCLA Cardiovascular Research Laboratory, Department of Medicine (Cardiology), David Geffen School of Medicine at University of California Los Angeles, Los Angeles, California, United States of America; University Paris Diderot-Paris 7, France

## Abstract

**Background:**

The first step in glucose metabolism is conversion of glucose to glucose 6-phosphate (G-6-P) by hexokinases (HKs), a family with 4 isoforms. The two most common isoforms, HKI and HKII, have overlapping tissue expression, but different subcellular distributions, with HKI associated mainly with mitochondria and HKII associated with both mitochondrial and cytoplasmic compartments. Here we tested the hypothesis that these different subcellular distributions are associated with different metabolic roles, with mitochondrially-bound HK's channeling G-6-P towards glycolysis (catabolic use), and cytoplasmic HKII regulating glycogen formation (anabolic use).

**Methodology/Principal Findings:**

To study subcellular translocation of HKs in living cells, we expressed HKI and HKII linked to YFP in CHO cells. We concomitantly recorded the effects on glucose handling using the FRET based intracellular glucose biosensor, FLIPglu-600 mM, and glycogen formation using a glycogen-associated protein, PTG, tagged with GFP. Our results demonstrate that HKI remains strongly bound to mitochondria, whereas HKII translocates between mitochondria and the cytosol in response to glucose, G-6-P and Akt, but not ATP. Metabolic measurements suggest that HKI exclusively promotes glycolysis, whereas HKII has a more complex role, promoting glycolysis when bound to mitochondria and glycogen synthesis when located in the cytosol. Glycogen breakdown upon glucose removal leads to HKII inhibition and dissociation from mitochondria, probably mediated by increases in glycogen-derived G-6-P.

**Conclusions/Significance:**

These findings show that the catabolic versus anabolic fate of glucose is dynamically regulated by extracellular glucose via signaling molecules such as intracellular glucose, G-6-P and Akt through regulation and subcellular translocation of HKII. In contrast, HKI, which activity and regulation is much less sensitive to these factors, is mainly committed to glycolysis. This may be an important mechanism by which HK's allow cells to adapt to changing metabolic conditions to maintain energy balance and avoid injury.

## Introduction

Upon entering the cell, glucose is phosphorylated to glucose-6-phosphate (G-6-P), which is used catabolically in glycolysis, or anabolically in glycogen synthesis and lipid synthesis via the pentose phosphate shunt. In both cases, this first step is catalyzed by hexokinases (HKs), which comprise a family of four isoforms. HKI and HKII are the most abundant isoforms, with HKI (“the brain HK”) ubiquitous in most tissues but especially brain and red blood cells [1], [2] where glycolysis plays a critical role in energy production. In contrast, HKII (“the muscle HK”) is found primarily in insulin-sensitive tissues such as adipocytes and adult skeletal and cardiac muscle, where it accounts for 80% of total HK activity [3]. At birth and after weaning, the latter tissues switch from expression of GLUT1 and HKI, to GLUT4 and HKII. The expression of GLUT4 and HKII coincides with the development of insulin sensitivity as muscle switches from a straight carbohydrate to a mixed fat-carbohydrate diet [4]. In adult muscle, fatty acids as well as glucose and glycogen are available as substrates to support oxidative metabolism [5].

G-6-P facilitates glycogen synthesis by reciprocally activating glycogen synthase (GS) and inhibiting glycogen phosphorylase (GP) [6], [7], and possibly by stimulating translocation of HK from mitochondria to the cytoplasm. In liver cells where glycogen synthesis is driven by glucokinase (GK or HKIV) rather than HKI, G-6-P stimulates glycogen synthesis by causing a redistribution of GK and GS to the cell periphery [8]. These data support the idea of compartmentalized metabolic channeling, with HKI feeding glycolysis, and HKII and HKIV feeding glycogen synthesis.

The idea that differing subcellular locations of HKI and HKII are important was postulated by Wilson when he stated that “the Type I isozyme bound to actively phosphorylating mitochondria facilitates introduction of glucose into glycolysis, with the final stages of glucose metabolism occurring in the mitochondria. In contrast, Type II and to some extent Type III isozymes serve primarily anabolic function to provide G-6-P for glycogen synthesis or lipid synthesis via the pentose phosphate pathway” (see review [9]). However, it was not clear at that time how independent was the anabolic function of the Type II isozyme and binding to mitochondria were related. Since then, Wilson and others [10], [11], [12], [13], [14], [15], [16], [17], [18] have shown that the interaction of HKs with mitochondria is not static, but is regulated by factors such as glucose, G-6-P and kinases such as Akt and GSK-3. Thus, a picture is emerging that HKII may play a dual role: channeling G-6-P into the glycogen and the pentose phosphate pathways when localized in the cytoplasm, and preferentially shuttling G-6-P to glycolysis and oxidative phosphorylation when bound to mitochondria [19]. In contrast, HKI generally facilitates glycolysis; although under some specific non-physiological conditions may contribute to glycogen synthesis [20].

HKI and HKII are inhibited allosterically by their product, G-6-P, and this sensitivity to G-6-P decreases when HKs are bound to mitochondria [21], [22]. Physiological levels of orthophosphate (Pi) counter the G-6-P inhibition of HKI [2], [23], [24], but not HKII. In fact, Pi may cause further HKII inhibition. Based on these observations, Wilson [9] suggested that “reciprocal changes in intracellular levels of G-6-P and Pi are closely associated with cellular energy status, and that the response of HKI to these effectors adapts it for catalytic function, introducing glucose into glycolytic metabolism. In contrast, HKII serves primarily anabolic functions.”

In the present study, we have expressed HKI and HKII tagged with YFP in Chinese Hamster Ovary (CHO) cells to track their subcellular location in real time and their mobilization in response to substrates. Concomitantly, we measured changes in intracellular glucose using a genetically-encoded intracellular glucose biosensor, FLIPglu-600 µM, which undergoes changes in FRET upon binding glucose [25] and assessed glycogen formation, also in live cells, using a glycogen targeting protein, PTG, tagged with GFP. In this study our goal was three-fold: 1) To further investigate the roles of HKI and HKII in directing the metabolic fate of glucose towards catabolic versus anabolic uses; 2) To assess how the metabolic roles of HKI and HKII are related to their subcellular localization; 3) To determine the signaling pathways regulating subcellular distributions of HKI and HKII. Our findings support the hypothesis that in response to changes in glucose, subcellular translocation of HKII dynamically directs the metabolic fate of glucose between catabolic (glycolysis) and anabolic (glycogen synthesis and pentose phosphate shunt) uses, while HKI remains associated with mitochondria to promote glycolysis. Factors such as G-6-P and Akt play a central role in the regulation of HKII activity and localization in response to changes in glucose.

## Results

### HK interaction with mitochondria and translocation to the cytosol

**Figure 1:**
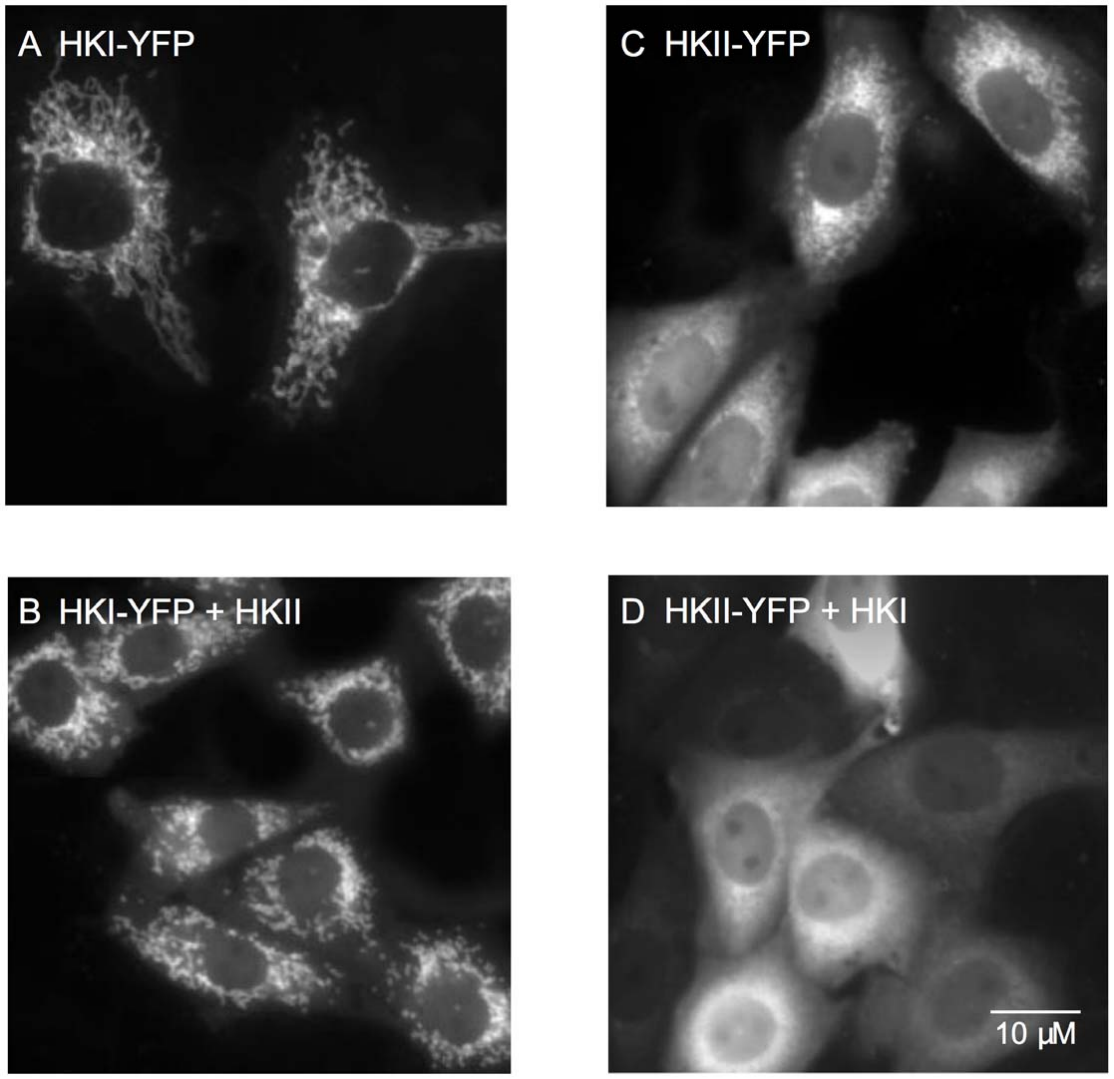
Subcellular distribution of HKI and HKII linked to YFP in CHO cells. In the left panel HKI-YFP is expressed alone (A) or with HKII (B) (ratio 1∶3). In this case HKI-YFP is bound to mitochondria and is not displaced by overexpressed HKII. In the right panel HKII-YFP is expressed alone and shows a mixed distribution between mitochondria and cytosol (C). In this case overexpression of HKI with HKII-YFP (ratio 1∶3) prevents HKII-YFP interaction with mitochondria (D). Similar subcellular distribution of HKI-YFP and HKII-YFP was observed in HEK293 cells.

To test the hypothesis that HKI interacts preferentially with mitochondria to facilitate entry of G-6-P into the glycolytic pathway, while HKII translocates into the cytosol to channel G-6-P into the glycogen formation pathway, we carried out two types of optical imaging experiments in CHO cells and HEK293 cells. In the first, we linked YFP to the C terminus of HKI or HKII (HKI-YFP and HKII-YFP) to track subcellular location. In the second, we used a genetically-encoded low affinity intracellular glucose sensor Flipglu-600 µM to monitor intracellular glucose metabolism. In Fig. 1A, data obtained with HKI-YFP demonstrates that HKI is bound to mitochondria with little or no fluorescence found in the cytosol. Co-labeling with HKI-CFP and MitoTracker-YFP (Fig. S1) supports the interpretation that HKI, even when overexpressed, is predominantly bound to mitochondria. In contrast, the distribution of HKII-YFP showed both mitochondrial labeling and diffuse fluorescence in the cytoplasm (Fig. 1C). To further characterize the interaction of HKs with mitochondria, we cotransfected HKI-YFP together with wild-type (wt) HKII (ratio 1∶3), and conversely, HKII-YFP together with wt HKI (ratio 1∶3). HKII did not displace HKI-YFP from mitochondria (Fig. 1B), while HKII-YFP was no longer associated with mitochondria in the presence of excess HKI (Fig. 1D). These data suggest that HKI has a strong affinity for a mitochondria-binding site which cannot be easily displaced by HKII. In contrast, HKII binding to mitochondria was much weaker and readily displaced by excess HKI.

### Effects of hexokinase overexpression in CHO cells transfected with GLUT1

Next, we investigated how HKI and HKII affect glucose metabolism by expressing a genetically encoded low affinity intracellular glucose sensor Flipglu-600 µM in native CHO cells. CHO cells express low levels of glucose transporters (GLUT), however, they have low rates of glucose uptake and metabolism compared to muscle cells [26]. Thus we overexpressed GLUT1 or GLUT4, to increase glucose uptake (Figs S2 and S3). GLUT overexpression both improved FRET signals and, facilitated glycogen synthesis, which is activated by high intracellular glucose. To characterize intracellular glucose handling, we used the GLUT inhibitor Cyto B (20 µM) to block GLUT-sensitive transmembrane glucose transport. We had previously shown that in native CHO cells, exposure to Cyto B prior to the addition of glucose fully blocked subsequent glucose uptake [26]. In contrast, Cyto B had almost no effect on the rate of glucose clearance following glucose removal (Fig. S3A [1] vs. Fig. S3A [2]). The latter observation suggests that glucose metabolism, rather than glucose efflux, dominates glucose clearance in native CHO cells with a slow rate of glucose transport (τd = 33 s). On the other hand, in CHO cells overexpressing GLUT1 (Fig. S3B [4]), with a high rate of glucose transport (uptake half-time 3.5 s), Cyto B markedly slowed the rate of glucose clearance upon glucose removal, indicating that in CHO cells overexpressing GLUT1, glucose efflux, rather than glycolysis, is the dominant factor regulating glucose clearance. Together, these observations indicate that in CHO cells overexpressing GLUT1 or GLUT4, glucose clearance is dominated by glucose efflux via GLUTs rather than glucose utilization by glycolysis. Thus, to study the metabolic fate of glucose in CHO cells overexpressing GLUTs, subsequent experiments were performed in the presence of Cyto B to block glucose efflux following glucose removal.

**Figure 2:**
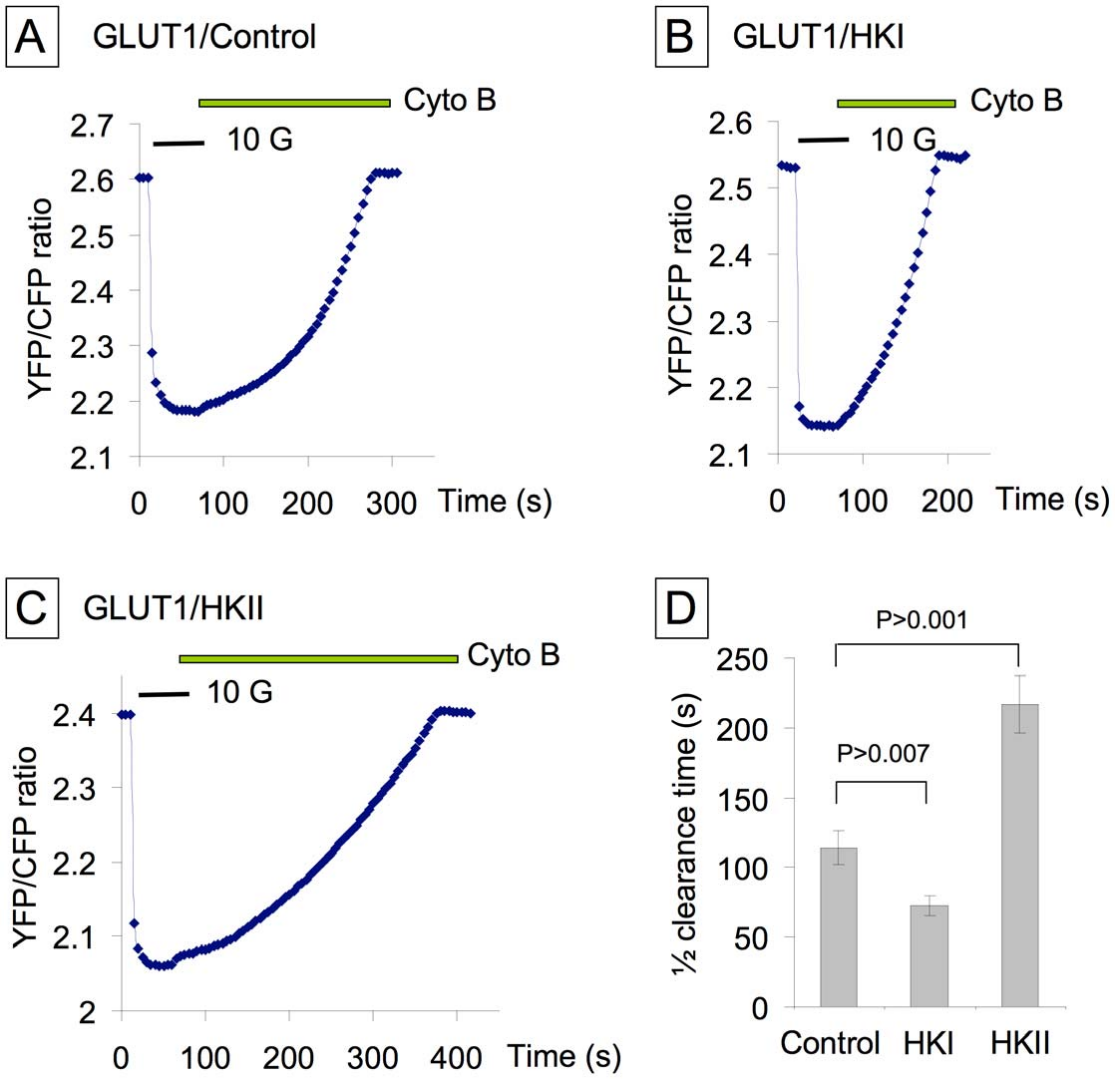
Effects of HKI and HKII on glucose utilization in CHO cells. These data illustrates how overexpression of HKI (Panel B) and HKII (Panel C) affects glucose clearance measured in the presence of CytoB. Comparison of A and B indicates that HKI increases the rate of glucose clearance and thus metabolism, while comparison of A and C demonstrates that HKII has the opposite effect and decreases this rate. Panel E and F show images similar to those in Fig. 1, which illustrate once more the interaction of HKI-YFP with mitochondria and the differential distribution of HKII-YFP, these differential distribution can be correlated with the glucose clearance data in B and C, respectively. The bar graph in D quantifies these data.

Data in Fig. 2, obtained with and without HK overexpression, show how expression of HKs alters the rate of glucose clearance during glucose removal in the presence of Cyto B. Panels B and D show that HKI overexpression decreased the average half-time of glucose clearance (t_1/2_) from 114±12 s to 72±7 s (n = 9) (Fig. 2D). In contrast, HKII overexpression (panels C and D) prolonged the half-time to 217+/−20 s (n = 9). Panels E and F show the distribution of overexpressed HKI or HKII in CHO cells. These data clearly demonstrate that increased HKI activity, which is primarily associated with mitochondria, increases glucose metabolism, while increased HKII activity, which is found at least in part in the cytosol, slows glucose metabolism.

To further assess the role of endogenous HKI and HKII in glucose clearance we transfected CHO cells with 30 to 40 nM of HKI or HKII siRNA. Knockdown of either HKI or HKII caused a decrease in the rate of glucose clearance (Fig S4). These results are consistent with our finding from RT-PCR analysis that the levels of endogenous HKI and HKII are very similar in CHO cells.

### Glycogen-derived G-6-P mediates glucose-dependent HK inhibition

**Figure 3:**
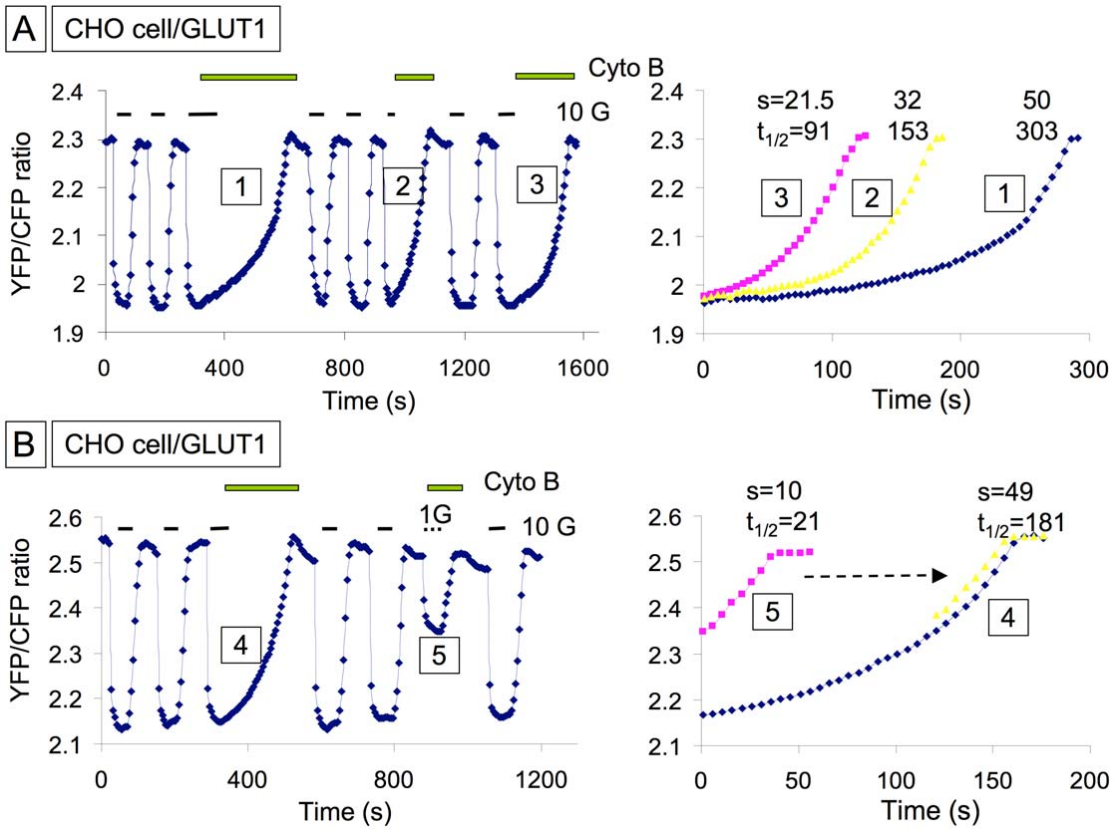
The time of exposure to extracellular glucose controls the rate of intracellular glucose clearance. Panel A illustrates how exposure to 10 mM extracellular glucose for 75 s (1), 30 s (2) and 50 s (3) affects intracellular glucose clearance. In the 3 cases CytoB was applied for 15 s prior to removal of extracellular glucose. The 3 rates are compared in the right hand side panel and show that the final rate of glucose clearance is similar in the 3 cases and that long exposure to extracellular glucose delays the clearance. In B a comparison of data obtained with 10 mM and 1 mM extracellular glucose show that application for up to two minutes of 1 mM glucose had no effect on the rate of intracellular glucose clearance, demonstrating that intracellular glucose must reach a threshold to induce this effect. The right hand side panel shows again that the final rate of glucose clearance measured in the presence of 10 mM glucose is similar to the maximum rate measure with 1 mM and the effect of high glucose is thus to delay clearance.

We hypothesized that when HKII is overexpressed, the prolonged t_1/2_ of glucose clearance following a glucose pulse is related to glycogen synthesis, which occurs during the glucose pulse. Thus, when glucose is removed at the end of the pulse, the cell mobilizes the newly synthesized glycogen for glycolysis, rather than metabolizing the residual intracellular glucose. This explanation is plausible only if glycogen mobilization also inhibits HK enzymatic activity, such that the conversion of residual intracellular glucose to G-6-P is suppressed. Since glycogenolysis involves production of G-6-P, which inhibits HKs [21], [22], we postulated that elevated G-6-P levels during glycogenolysis might be responsible for inhibiting HK activity, so that intracellular glucose clearance is delayed. Consistent with this hypothesis, Fig. 3A shows after a 30 s exposure to glucose (Fig. 3A [2]), glucose clearance upon removing extracellular glucose (in the presence of Cyto B) was rapid, and occurred at a rate similar to that observed in the absence of GLUT overexpression. However, after a 75 s exposure to 10 mM glucose (Fig. 3A [1]), to allow more time for glycogen synthesis, glucose clearance by metabolism was delayed, occurring very slow initially, followed by rapid clearance similar to that after the short exposure to glucose. The t_1/2_ increased on average from 88±14 s after a 30 s exposure to glucose, to 285±29 s (n = 15) after a 75 s exposure. In contrast, exposure to 1 mM extracellular glucose for several minutes, which increased intracellular glucose level by less than half compared to 10 mM glucose, did not cause a delay in glucose metabolism (Fig. 3B [4] and [5]).

**Figure 4:**
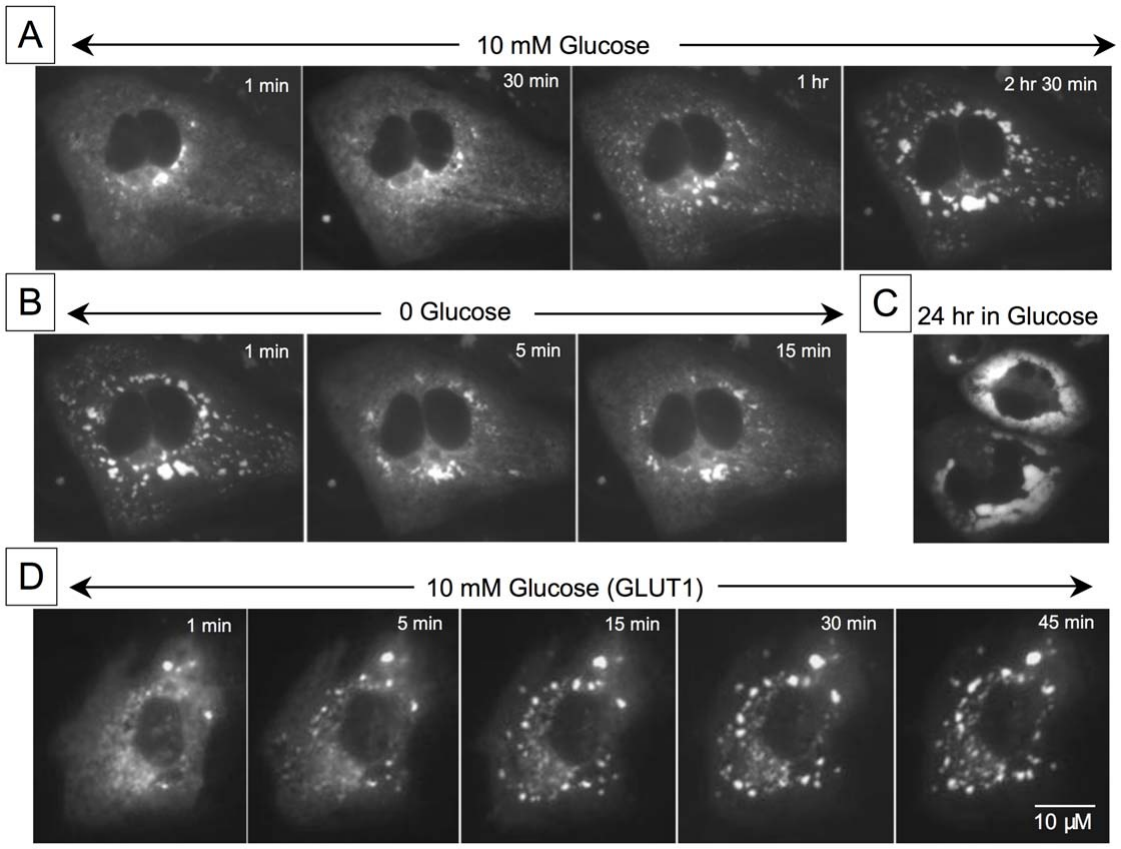
Effects of GLUT1 overexpression on glycogen synthesis and breakdown in CHO cells. The rate of glycogen formation without (A) and with (D) overexpression of GLUT1 and the rate of glycogen degradation (B) was studied using a protein targeted to glycogen linked to YFP (PTG-YFP). In Panel A and D the cells were incubated in the absence of glucose for 2 to 3 hours prior to beginning cell imaging. This incubation in the absence of glucose was carried out to deplete preformed glycogen. Without GLUT1 overexpression glycogen build up was slow, occurring over several hours. This build up proceeded for up to 24 h filling almost completely the cell in some cases (C). With GLUT1 overexpression the rate of glycogen synthesis increased and glycogen deposits could be observed 5 min after exposure to 10 mM glucose outside. It is interesting to note that glycogen deposition occurred at least initially near the nucleus where mitochondria aggregate, suggesting that G-6-P generated by glycogen degradation may be directly fed on to mitochondria.

To test this hypothesis further, we imaged glycogen stores directly by using probes linking either mCherry or GFP to PTG and G_L_, both of which are part of the family of glycogen targeting subunits of PP-1 [27]. CHO cells were transfected with one of these probes, either with or without GLUT1 co-transfection. One day after transfection, cells were incubated for 2 to 3 hours in the absence of glucose to deplete glycogen stores. In the absence of GLUT1 overexpression, some CHO cells exhibited dim homogenous fluorescence, while others had a few small and bright punctuate deposits (Fig. 4A). After re-addition of glucose (10 mM) for 30–60 min, the number, size and brightness of small glycogen deposits began to increase. This phenomenon intensified over 24 hrs, until the deposits fused and partially filled the cell (Fig. 4C). After a two hour exposure, removal of glucose resulted in a rapid disappearance of glycogen deposits (Fig. 4B). With overexpression of GLUT1, the rate of appearance of glycogen dramatically increased, such that small deposits were observed within a few minutes of exposure to 10 mM glucose (Fig. 4D). Thus, in CHO cells overexpressing GLUT1, the rates of glycogen synthesis and depletion are rapid enough to plausibly support our hypothesis that relatively short glucose pulses could induce enough glycogen synthesis to progressively delay the t_1/2_ of glucose clearance following glucose removal.

**Figure 5:**
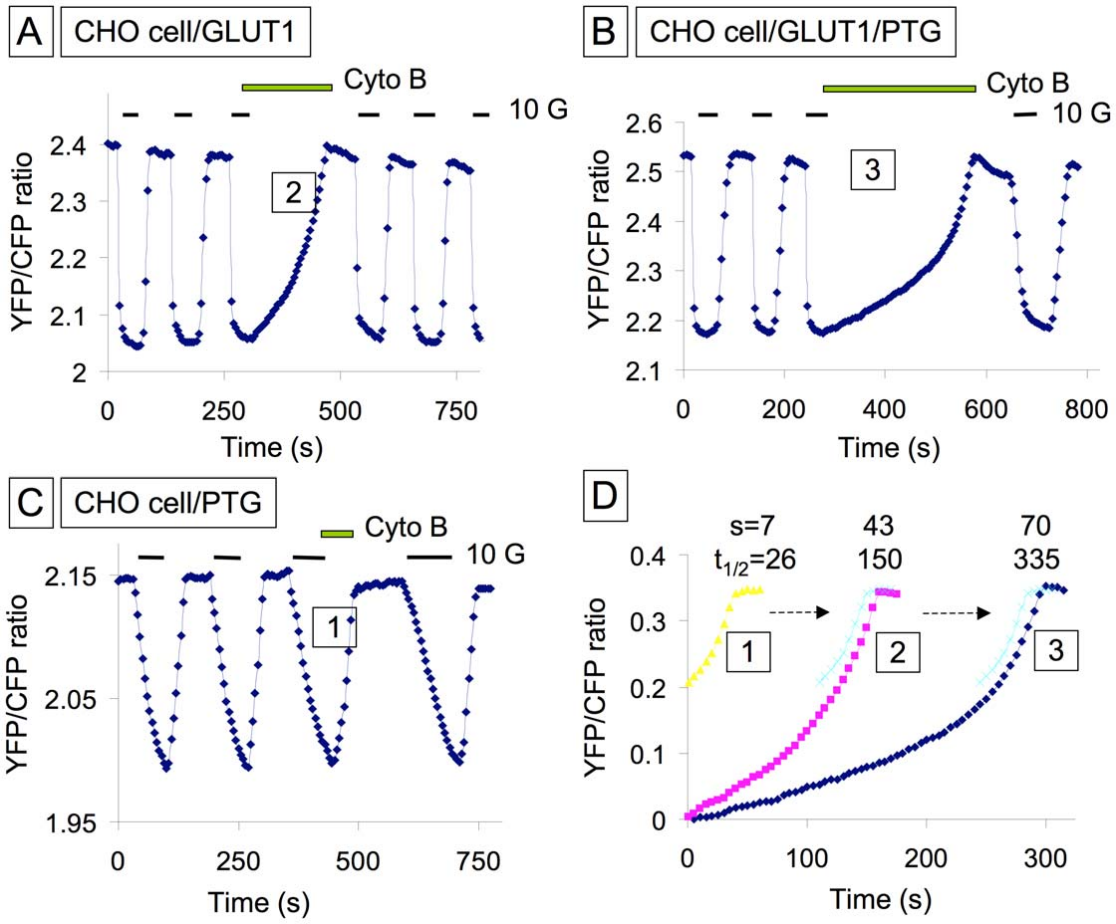
Overexpression of PTG delays glucose clearance. Panels A and B obtained with CHO cells overexpressing GLUT1 illustrate how exogenous PTG delays glucose clearance. In both cases the cells were exposed to extracellular glucose for 50 s. Comparison of data in Panel B (overexpressed GLUT1) and C (no GLUT1 overexpression) illustrate the lack of effect of exogenous PTG in the absence of GLUT1 overexpression, supporting the hypothesis that GLUT1 is necessary for rapid build up of the glycogen store and inhibition of glucose utilization. Compilation of traces in D illustrates once more that the final rate of glucose clearance is similar in all cases and that increased glycogen synthesis only delays glucose utilization.

The effects of PTG overexpression on the t_1/2_ of glucose clearance following glucose removal are shown in Fig. 5. In CHO cells transfected with PTG alone, PTG had no effect on the t_1/2_ of glucose clearance (Fig. 5C), even after prolonged exposure to glucose, which we attributed to intracellular glucose never reaching an adequate level to induce glycogen synthesis (as indicated by a low FRET ratio). However, in CHO cells transfected with both GLUT1 and PTG, the lag phase was pronounced after even a brief exposure to 10 mM glucose (Fig. 5B). Thus, PTG, which stimulates glycogen synthesis by facilitating the interaction of the regulatory enzyme PP-1 (protein phosphatase-1) with GS, GP and phosphorylase kinase, leads to greater glycogen synthesis during brief exposures to 10 mM glucose. According to our hypothesis, this elevates G-6-P levels for longer after glucose removal and delays glucose clearance.

In summary, these data are consistent with removal of glucose causing a switch from glycogen synthesis to glycogen breakdown, thereby increasing G-6-P which blocks the utilization of glucose by mitochondria-bound HK, in effect diverting G-6-P to glycolysis.

### Regulation of the metabolic fate of glucose by the subcellular distribution of HK

**Figure 6:**
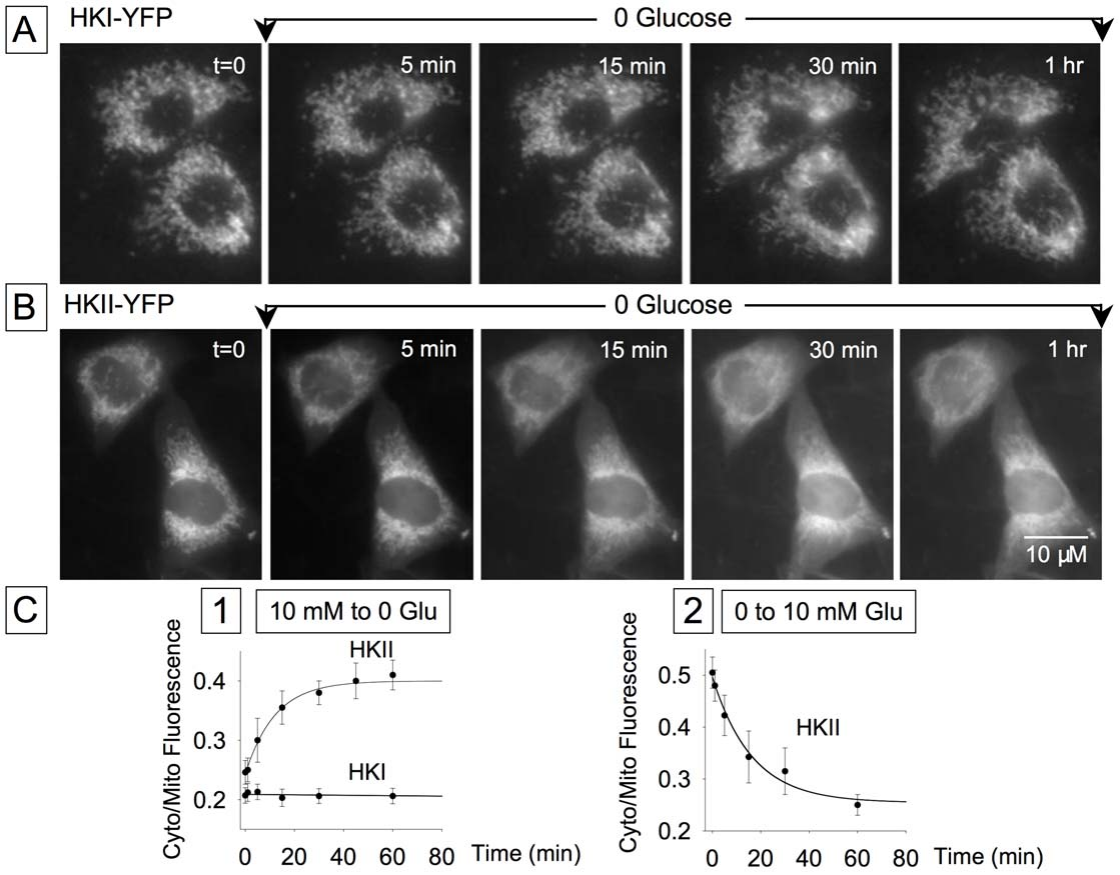
Removal of glucose causes translocation of HKII, but not HKI, into the cytosol. Images in Panel A obtained with HKI-YFP expressed in CHO cells show that removal of glucose did not affect HKI interaction with mitochondria. In contrast, HKII begun to dissociate from mitochondria 5 min after glucose removal (B). Panel C shows the rates measured as ratio of fluorescence intensity obtained from intracellular domains without and with mitochondria. In almost all cases the region without mitochondria was selected at the cell periphery and that with mitochondria was selected near the nucleus. C1 is a comparison of the rates of HKI and HKII dissociation in response to glucose removal. C2 shows the rate of HKII reassociation with mitochondria after glucose readdition.

The observations so far indicate that HK1 overexpression affects catabolism, accelerating the t_1/2_ of glucose clearance, whereas HKII overexpression is anabolic, delaying the t_1/2_ by promoting glycogen synthesis, with subsequent inhibition of HK by glycogenolysis when extracellular glucose is removed. To determine how these metabolic roles are related to the subcellular localization of HKI and HKII, we studied the effects of extracellular glucose on subcellular HK distribution in CHO cells overexpressing HKI or HKII linked to YFP. Whether the cells were incubated in the presence or absence of glucose (10 mM), HKI-YFP always remained associated with mitochondria, and addition or removal of glucose had no effect on its location (n = 10) (Fig. 6A). In contrast, the subcellular distribution of HKII-YFP was sensitive to extracellular glucose. In the presence of glucose, a large fraction of HKII was bound to mitochondria, and upon removal of extracellular glucose, HKII rapidly translocated to the cytosol with a time constant averaging 8.5 min (n = 12) (Fig. 6B and 6C1). This effect was fully reversible, such that upon re-addition of glucose, HKII re-associated with mitochondria with a time constant of 15.5 min (n = 4) (Fig. 6C2). These data support the hypothesis that HKI binds strongly to mitochondria, while the distribution of HKII between cytoplasm and mitochondria is labile, dynamically regulated by glucose availability.

**Figure 7:**
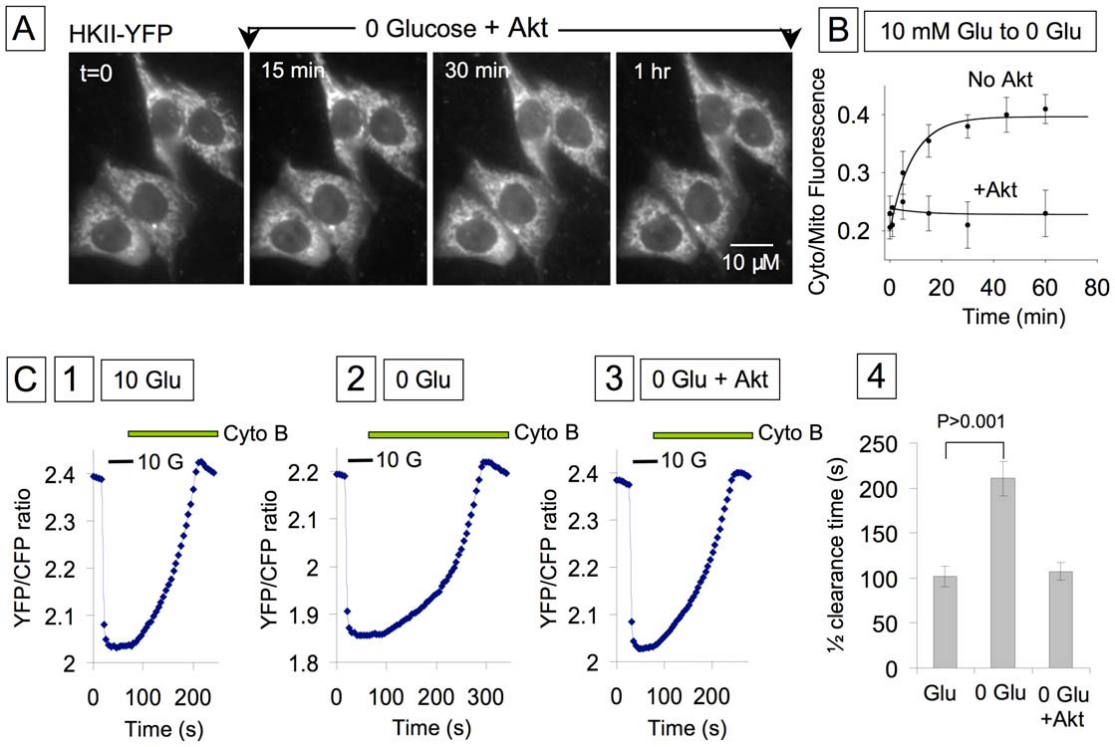
Constitutively active Akt prevents the effect of glucose removal on HKII dissociation from mitochondria. Images in (A) obtained with overexpression of a constitutively active Akt illustrate how Akt prevents HKII dissociation evoked by removal of glucose. Panel B shows a quantification of this effect, comparing the rate of HKII dissociation from mitochondria in response to glucose removal in the presence and absence of exogenous Akt. Data in Panel C show how glucose clearance is affected by preincubation in the absence of glucose and how constitutively active Akt prevents this effect. Previous data (Fig. 6B) have shown that HKII dissociates from mitochondria 15 to 30 min after removal of extracellular glucose; comparison of traces C1 and C2 (in C2 cells were incubated for 30 min in the absence of glucose) indicates that this effect is accompanied by a decrease in glucose clearance. Thus, glucose-induced HK dissociation slows the rate of glucose utilization. The recording in C3 obtained with cells overexpressing constitutively active Akt indicates that Akt prevents the decrease in glucose clearance induced by incubation in the absence of glucose for 30 min. This effect is very likely related to the effect of Akt on HKII translocation. The bar graph in C4 quantifies the effects of glucose removal and Akt on glucose clearance.

To evaluate the functional consequences of HKII redistribution on glucose metabolism, cells were subjected to a 20 to 30 min pre-incubation in the absence of glucose to maximize dissociation of HKII from mitochondria (Fig. 6B), and compared to cells with glucose present throughout (in which HKII remained mostly associated with mitochondria). Preincubation in zero glucose prolonged the t_1/2_ of glucose clearance from 102±11 s to 210±19 s (n = 7) (Fig. 7C2 and C4). This finding demonstrates that the subcellular distribution of HKII strongly influences its ability to promote anabolic use of glucose for glycogen synthesis.

Since Akt facilitates HK interaction with mitochondria [14], we examined whether translocation of HKII from mitochondria to cytoplasm in response to zero glucose pre-incubation was suppressed by overexpression of constitutively activate Akt. With Akt overexpression, the mixed pattern of mitochondria-bound and cytosol-associated HKII-YFP did not change (Fig. 7A), and prolongation of the t_1/2_ of glucose clearance by zero glucose preincubation was abolished 107.0±9.5 s versus 210±19 s (n = 8) with and without Akt) (Fig. 7A and Fig. 7C3 and C4).

Thus, both glucose and Akt signaling promote the binding of HKII to mitochondria, favoring glucose catabolism over glycogen synthesis.

### Role of G-6-P in regulating HK activity and subcellular distribution

**Figure 8:**
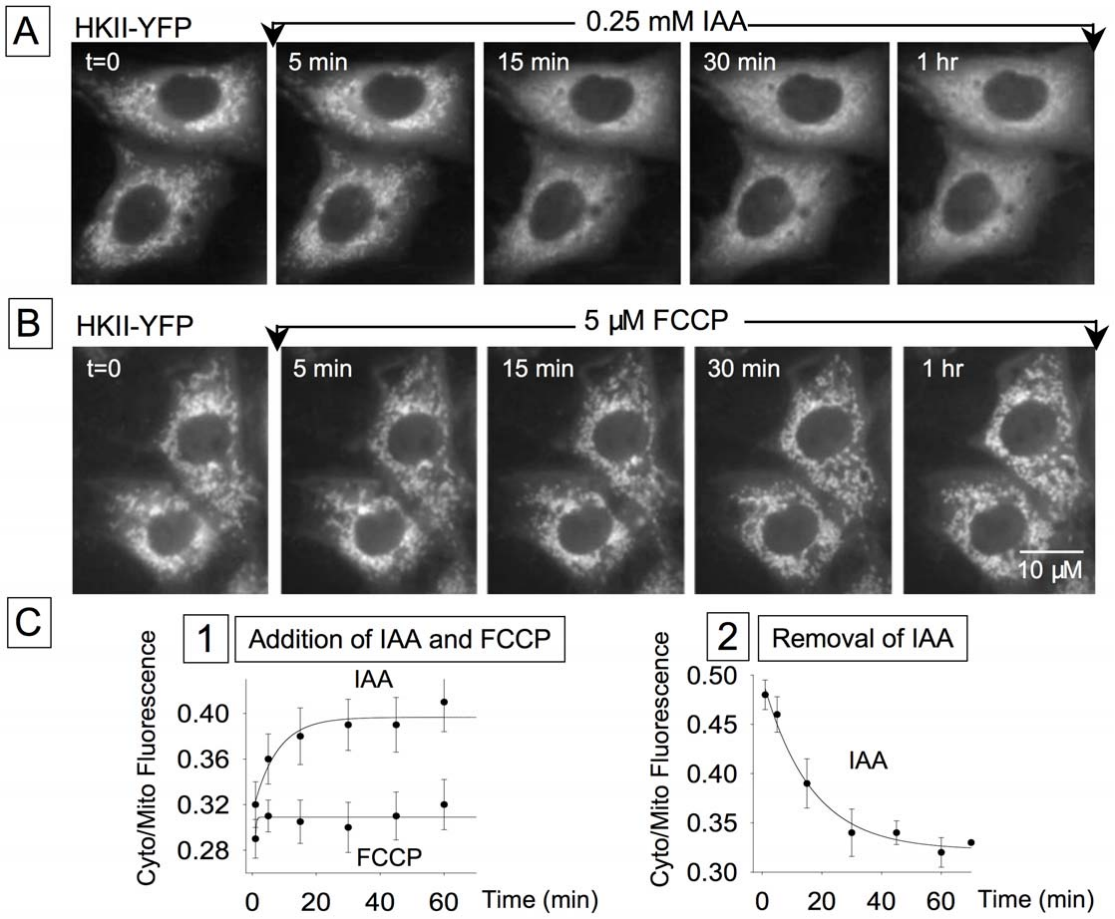
Rates of association and dissociation of HKI and HKII in response to glucose, IAA and FCCP. IAA, but not FCCP, facilitates HKII dissociation from mitochondria. Images in Panel A obtained with HKII-YFP expressed in CHO cells show that addition of the glycolysis inhibitor, IAA, in the presence of 10 mM glucose causes HKII dissociation from mitochondria within 5 to 15 min. In contrast, addition of FCCP, which depletes mitochondrial ATP, had no effect on HKII interaction with mitochondria (B). These data indicate that G-6-P, rather than ATP mediates the effect of glucose removal on HKII translocation. Panel C shows the rates measured as ratio of fluorescence intensity obtained from intracellular domains without and with mitochondria. C1 is a comparison of the rates of HKII dissociation from mitochondria in response to IAA and FCCP addition in the presence of 10 mM glucose outside. C2 illustrates the rate of reassociation HKII with mitochondria upon removal of IAA.

We have hypothesized that HKII translocation to the cytosol delays the t_1/2_ of glucose clearance when glucose is removed because newly synthesized glycogen is mobilized and in the process keeps G-6-P levels elevated, suppressing HK activity until glycogenolysis ceases. We tested this hypothesis using both direct and indirect approaches. First, in the presence of glucose, we applied iodoacetate (IAA, 0.25 mM) to elevate G-6-P levels by inhibiting glycolysis. As shown in Fig. 8A and C1, IAA caused translocation of HKII into the cytosol, in the continued presence of 10 mM glucose, with a time constant of 7.5 s (n = 10). This effect was fully reversible and HKII re-associated with mitochondria with a time constant of 16 s (n = 9) following removal of IAA (Fig. 8C2). To rule out ATP depletion, rather than G-6-P accumulation, as the cause of HKII translocation, we also tested the mitochondrial uncoupler FCCP (5 µM) to deplete mitochondrial ATP without elevating G-6-P levels. In 6 cells, FCCP had no effect on HKII translocation (Fig. 8B). These data support our hypothesis that the effect of glucose on HKII translocation and inhibition is mediated via G-6-P.

**Figure 9:**
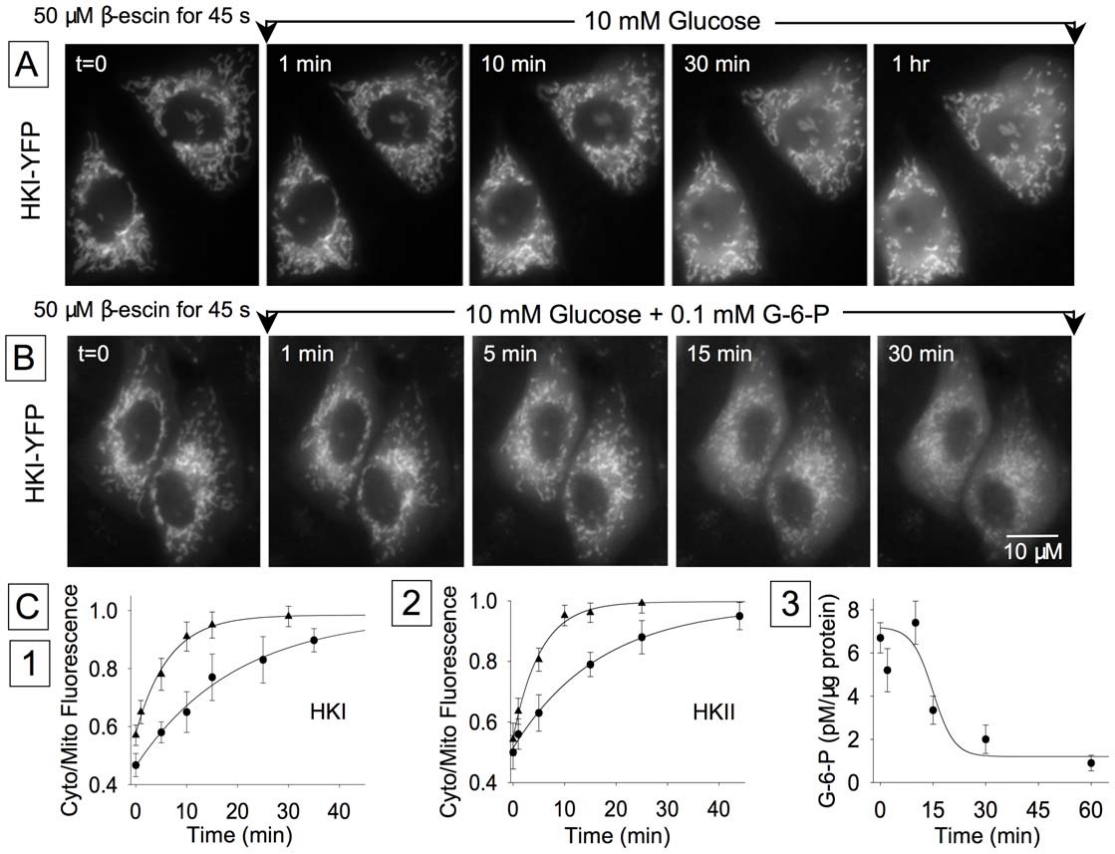
HKs dissociation from mitochondria in permeabilized cells. Panels A and B illustrate the spontaneous dissociation of HKI-YFP in the absence and presence of G-6-P, respectively, after cell membrane permeabilization with 50 µM β-escin. Panel C1 and C2 show that HKI and HKII dissociate for mitochondria at very similar rates, and G-6-P increases these rates by approximately 4 fold (20 min vs. 5 min). The triangles represent measurements obtained with G-6-P (100 nM) and the circles are for values obtained in the absence of G-6-P. Panel C3 shows the change in intracellular G-6-P levels following glucose removal.

To obtain further evidence that G-6-P causes dissociation of HKs from mitochondria, we used permeabilized CHO cells overexpressing HKI-YFP or HKII-YFP. Both HKI and HKII spontaneously dissociated slowly from mitochondria when the plasma membrane was permeabilized using 50 µM β escin (Fig. 9), with time constants of 19.5 min (n = 6) and 17 min (n = 5) (Fig. 9C), respectively although the difference was not significant. Furthermore addition of 100 nM G-6-P at the time of cell membrane permeabilization accelerated the rates of both HKI and HKII dissociation (τ = 6.2 min (n = 4) for HKI and τ = 5.5 min (n = 5) for HKII). Our data obtained with permeabilized cells corroborate previous findings that G-6-P displaces both HKI and HKII from isolated mitochondria [17]. However when combined with our data obtained from intact cells, indicate that an intracellular factor is present in intact cells that prevents the dissociation of HKI induced by G-6-P in isolated mitochondria. Importantly, our data suggest that, in the absence of any factor other than G-6-P the two HKs have similar affinity for mitochondrial membrane and dissociate at similar rates.

Finally, we directly measured intracellular G-6-P levels following removal of 10 mM glucose in CHO cells transfected with GLUT1. Upon removal of glucose, G-6-P levels remained elevated for about ∼10 min and then decreased in a sigmoidal fashion with 50% decay reached after ∼15 min (Fig. 9C3 each data point in the graph represents an average of 8 to 26 values). Together, these data corroborate our glucose metabolism measurements, and support the hypothesis that upon glucose removal G-6-P elevation inhibits glucose phosphorylation by hexokinases. The decay in G-6-P that follows, accounts for the gradual reactivation of hexokinase activity and resumption of glucose phosphorylation.

## Discussion

Catabolic and anabolic glucose utilization are both directed by hexokinases, which channel G-6-P to glycolysis or glycogen and lipid synthesis. While HKs coexist in many cell types, cells that generate glycogen in response to insulin, such as adult muscle, express primarily HKII, whereas cells that rely primarily on glycolysis for energy production, such as the brain, express high levels of HKI. The specificity of these enzymes is not related to functional differences, since both phosphorylate glucose to G-6-P, but may instead depend on subcellular location, reflecting spatial compartmentalization of glucose metabolism. Thus, it has been suggested that the association of HKI with mitochondria channels glucose to the glycolytic pathway, whereas HKII, when translocated to the cytoplasm, controls glycogen formation [9]. Our results using live cell imaging to track the subcellular distributions of HKI and HKII provide direct support for this hypothesis.

### Glycogen breakdown generates G-6-P that inhibits HK enzymatic activity

Our data show that HKI is strongly associated with mitochondria, while HKII dynamically translocates between mitochondria and the cytoplasm. This differential localization is associated with a fast rate of glucose utilization upon removal of extracellular glucose in cells expressing HKI, but inhibition of glucose utilization in cells expressing high levels of HKII. We propose that cytosolic HK II channel G-6-P towards the glycogen synthesis pathway during exposure to 10 mM glucose, such that upon glucose removal, mobilization of the newly-synthesized glycogen regenerates G-6-P (or another glycolytic intermediate) which inhibits HK activity. This phenomenon is primarily observed in cells with cytosolic HKs that favor glycogen synthesis. In cells with mitochondria-associated HKs, G-6-P is channeled towards the glycolytic pathway and there is no G-6-P-induced inhibition. The following evidence supports these hypotheses: 1) Conditions which favor glycogen formation, such as PTG overexpression and long exposure to glucose, enhances the duration of the inhibitory phase; 2) The effect of glucose is only observed with overexpression of GLUT1 in CHO cells, which raises intracellular glucose enough to rapidly stimulate glycogen synthesis. Indeed, only with overexpression of GLUT1 can glycogen synthesis be stimulated by glucose within minutes. Without GLUT1 overexpression, significant glycogen synthesis requires more than 30 min of glucose exposure and there is no HK inhibition upon glucose removal; 3) Akt, which facilitates HKII interaction with mitochondria, reduces the inhibitory phase and removal of glucose, which causes HKII dissociation from mitochondria, enhances the inhibitory phase.

### G-6-P regulates HK enzymatic activity as well as subcellular localization

In HKI, the C terminal domain binds ATP and exhibits catalytic activity, while the N terminal domain facilitates HK interaction with the mitochondria outer membrane. Both domains bind glucose and G-6-P synergistically. Binding of G-6-P to the C and N terminal domains inhibits enzymatic activity and causes dissociation from mitochondria either directly or via allosteric interaction between the two domains. Pi prevents HKI inhibition and dissociation by competing with G-6-P at the N terminal domain [2], [17], [18], [19], [23], [24], [28]. On the other hand, Pi does not bind to HKII and both the N- and C- domains exhibit catalytic activity. We have shown that HKII dissociation from mitochondria is insensitive to FCCP, which induces ATP depletion, but is facilitated by the glycolytic inhibitor IAA, which elevates G-6-P. These results indicate that the effect of glucose on HKII translocation is mediated by G-6-P and not by ATP. Thus, ATP regulates the catalytic activity of HKs, but not their association with mitochondria. The lack of effect of glucose removal and IAA on HKI association with mitochondria supports the hypothesis that Pi competes with G-6-P to strengthen the interaction with mitochondria. We have shown that upon plasma membrane permeabilization, both HKI and HKII dissociate from mitochondria with a time constant of approximately 20 min, which is accelerated 4-fold by 100 nM G-6-P. These data are consistent with results obtained with isolated mitochondria demonstrating that G-6-P facilitates dissociation of both HKI and HKII from mitochondria. Furthermore, our finding that HKI does not dissociate from mitochondria upon glucose removal in intact cells supports the hypothesis that an additional factor, perhaps Pi, prevents the effect of G-6-P on HKI in intact cells.

Phosphorylation of residue T473 by Akt in the linker region between the N and C terminal domain of HKII may facilitate its interaction with mitochondria [14]. It has been proposed that this effect of Akt requires the presence of glucose, based on the observation that the protective effect of Akt against apoptosis, which is mediated by HKs, requires glucose [13]. Our results demonstrate that Akt does indeed regulate the interaction of HKII with mitochondria, but its main effect is to prevent dissociation from mitochondria upon glucose removal. To account for these results, we suggest that Akt phosphorylates mitochondria-bound HKII, thereby preventing the ability of G-6-P to reduce HK mitochondrial interaction.

### Physiological relevance of preferential expression of HKI and HKII

In most native tissues such as muscle, liver and fat, hexokinases coexist, but in muscle HKI is most abundant in fetal tissues, while HKII represent 80% of total hexokinase activity in adult muscle [3], [4]. In adult muscle the increased expression of HKII is associated with increased expression of GLUT4 and the development of insulin sensitivity. In this context, HKII is both cytosolic as well as bound to mitochondria. In light of our data, it is interesting to speculate how this switch in HK and GLUT affects glucose metabolism and cell function. In adult muscle when extracellular glucose is high, for instance during a meal, G-6-P is channeled towards both glycogen synthesis and glycolysis, so that G-6-P levels remain low. However, between meals, when extracellular glucose decreases and more importantly when insulin is no longer released by pancreatic β cells, GLUT4 is internalized and intracellular glucose drops dramatically. The ensuing G-6-P production resulting from glycogen breakdown inhibits mitochondria-bound HKII-mediated glycolysis within seconds. Elevated G-6-P also causes dissociation of HKII from mitochondria further inhibiting glucose utilization. G-6-P generated by glycogen breakdown is then channeled through the glycolytic pathway to be further metabolized. In contrast, in fetal tissue with high level of HKI and GLUT1 most HKs are bound to mitochondria and the low levels of HKII found in the cytosol only generates little glycogen during meals. Moreover, mitochondria-bound HKI being poorly sensitive to G-6-P it follows that the low level of G-6-P produced in between meals will have minimal effect on HK activity and translocation. Finally, GLUT1 insertion into the plasma membrane is not regulated by insulin, as a result glucose uptake and intracellular glucose remain high even when blood glucose decreases, further preventing glycogen degradation. These properties ensure that glycolysis is the main source of energy in these fetal tissues.

Such difference between HKI and HKII would not only affect differentially glucose metabolism in fetal and adult muscle, but also cell fate. There is a large body of evidence that mitochondria-bound HKs protect against apoptosis via a direct interaction with VDAC, which prevents the mitochondrial permeability transition pore opening. Alternatively, the anti-apoptotic effect of mitochondria-bound HKs may require glucose metabolism [12], [29]. It follows that during stress fetal cells preferentially expressing HKI may cope better than adult cells expressing HKII as HKI remains associated with mitochondria, thus maintaining glycolysis activity and stability of VDAC in a low permeability state.

In summary, our data suggest that in cells that preferentially express HKII and GLUT4, such as adult muscle, removal of glucose causes the glycogen pathway to switch from synthesis to degradation. The ensuing elevation of G-6-P inhibits and then displaces HKII from mitochondria, causing the cell to switch from glucose to glycogen utilization as a source of energy. In contrast, in cells like neonatal muscle cells, which express preferentially HKI and GLUT1, the strong association of HKI with mitochondria implies that the cells primarily rely on glucose utilization as a source of energy and glycogen has a minor role. In this case, the strong association of HKI with mitochondria may also protect against metabolic stress.

## Materials and Methods

### Solutions and experimental techniques

The bath solution for cell imaging consisted of (in mM) 140 NaCl, 5 KCl, 1.1 MgCl_2_, 2.5 CaCl_2_, 10 HEPES, with the pH adjusted to 7.2 with KOH. Glucose was added to this solution and NMDG was added to maintain the solution's osmolarity in the absence of glucose. Solutions were perfused directly over the cells using a gravity fed eight way perfusion device (Warner Instruments, Hamden, CT) with electrically controlled solenoids (The Lee Company, Westbrook, CT). Input and output of solution volumes to the recording chamber (glass bottomed Petri dish) were equilibrated to maintain constant flow rates and pressures within the recording chamber. Experiments were carried out at room temperature (25°C). Cytochalasin B (Cyto B) and other chemicals were purchased from Sigma-Aldrich (St Louis, MO).

### Molecular biology and cell culture

We obtained the FLIPglu-600 µM cDNA from Dr. W.B. Frommer, GLUT from Dr. D. Abel, GLUT4-GFP from Dr. S. Cushman, rat HKI and HKII from Dr. J. Wilson, PTG from Dr. M Brady and GL-GFP and PTG-GFP from Dr. J. Guinovart. Fusion of PTG or GL to mCherry was carried out by subcloning into the mCherry N-1 vector. Constitutively active Akt was obtained from Dr. W. Sellers (Addgene plasmid 9009). Fusion of rat HK's to YFP was accomplished by inserting a Bam H1 site at the last amino acid of the coding sequence and subcloning into a modified pEYFPN-1vector (Clontech, Mountain View, CA). The modified pEYFPN-1 carried the mutations Q86K and A206K. Similarly, GLUT1-GFP was constructed by inserting a Kpn site at the last amino acid of the coding sequence and subcloning into pEGFPN-1 vector (Clontech, Mountain View, CA). All constructs were subcloned into the mammalian expression vectors utilizing the CMV promoter. CHO cells were transfected with 1 µg DNA plus 2.5 µl lipofectamine 2000 (Invitrogen, Carlsbad, CA) per 1.5 cm^3^ Petri dish. Expression of FLIPglu-600 µM was sufficiently high after 24 h to perform FRET experiments. Cells were cultured in DMEM high (25 mM) glucose medium supplemented with 10% (v/v) fetal bovine serum (FBS), penicillin (100 units/ml), streptomycin (100 units/ml) and 2 mM glutamine and divided once a week by treatment with trypsin.

### siRNA-mediated depletion and quantitative PCR

CHO cells (0.5–2×10^6^) were transfected with 30 to 40 nM siRNAs using lipofectamine 2000 reagent (Invitrogen, Carlsbad CA). Cells were used for experiments 2 days after transfection. The sequences of siRNAs (DsRNA from IDT, San Diego, CA) were:

HKI AACGTGAATCCCACAGGTAACTTCTTG and CGGATGTCTTCTAATGATCCATCGTC

HKII GTATCCAATTCAATAGTTACATCCCTC and CTTTGGTTTCCTTTGCTTAACATCCCA

RT–PCR analysis. Purified RNA from CHO cells (2–8×10^6^) was isolated using the RNAeasy Kit (Qiagen, Valencia, CA). First-strand cDNA synthesis was primed with oligo (dT) (Superscript III kit, Invitrogen, Carlsbad, CA). Real-time PCR was performed using an iCycler IQ5 (Bio-Rad, Hercules, CA) with the iQ SYBR Green Supermix (Bio-Rad, Hercules, CA). The cDNA levels were normalized to glyceraldehyde 3-phosphate dehydrogenase (GAPDH) or actin. Values shown in Supplementary Table S1 are C_T_ test – C_T_ GAPDH. Sequences of the primers were:

HKI GAAGAATGGCCTCTCCCGGG and GCCATGCACGATGTTCTCTGG

HKII AGAGCATCCTCCTCAAGTGGAC and ACCAAGTGCAGAAGGTTGACCA

GAPDH GCGCCAGCATCACCCCATTTGATG and GGTCGGCGTGAACGGATTTGGCCG

Actin TGGCATCCACGAAACTACAT AND TGGTACCACCAGACAGCACT

### Cell membrane permeabilization

The cells were permeabilized with 50 µM β-escin dissolved in an intracellular-type solution containing 140 mM KCl, 5 mM NaCl, 0.5 mM MgCl_2_, 100 nM Ca^2+^, 5 mM glucose and 1 mM DTT. To monitor cell membrane permeabilization we expressed the fluorophore CFP together with HKx-YFP. After exposure to β-escin for about 45 s CFP leaked slowly out of the cell over a period of 30 to 45 min, indicating cell membrane permeabilzation. At the same time HKs begun to slowly dissociate from mitochondria.

### FRET imaging

All cells were imaged live without fixation. Images (16-bit) were acquired using a Nikon Eclipse TE300 microscope fitted with a 60× (N.A. 1.4) oil immersion lens (Nikon, Melville, NY) and equipped with a filter cube comprising a CFP bandpass excitation filter: 436/20b, together with a longpass dichroic mirror: 455DCLP (Chroma Technology Corp, Rockingham, VT). LED's (Lumileds; San Jose, CA) were used as light sources: one emitting at 455±20 nm (royal blue) and the other emitting at 505±15 nm (cyan). LED's and camera exposure were controlled by MetaFluor Imaging 6.1 software (Molecular Devices Corp., Sunnyvale, CA).

Ratiometric FRET measurements were performed by simultaneously monitoring CFP and YFP emissions of the sample when excited at the wavelengths for CFP (royal blue LED), as described previously [26]. The ratio between YFP and CFP emission were measured online in real time using MetaFluor Imaging software. For analysis, background light intensity was subtracted from the individual YFP and CFP emission. YFP and CFP images were acquired simultaneously using a Dual View image splitter (Optical Insights, Tucson, AZ) equipped with a 505 nm long-pass dichroic filter to separate the CFP and YFP signals, a CFP emission filter (480/30) and a YFP emission filter (535/40). Superposition of the CFP and YFP images was carried out using the imaging software. Images were captured with a Cascade 512B digital camera (Photometrics, Tucson, AZ). Exposure times were optimized in each case but varied between 300–500 ms and were recorded at a constant rate for each cell between 0.2–0.33 Hz. Many experiments lasted more than one hour leading to a slow drift in the FRET ratio baseline in some cases. In most figures an initial drift in FRET ratio was corrected using exponential curve fitting.

In most cases changes in the FRET ratio measured upon addition of glucose could be fitted to a single exponential. FRET ratio recovery following glucose removal could not be fitted to a single exponential due to a lag in glucose clearance following removal of extracellular glucose. In this case the rate of glucose clearance was satisfactorily fitted to a sigmoidal function y = y_0_+a/1+e−(x_0_/s), where ×0, an estimate of the time requires to reach half of the change in FRET ratio, accounts for the lag period, while s, an estimate of the rate of change is more or less independent of the lag period.

Translocation imaging of HKs between mitochondria and cytoplasm was quantified as ratio of light intensity between two intracellular regions of interest with and without a high concentration of mitochondria. Background light was subtracted in both cases. Images were acquired using an Olympus IX70 inverted microscope (Olympus America Inc., Center Valley, PA) fitted with an Olympus plan apo ×60. 1.4 N.A. oil immersion objective and a cooled CCD camera (Model Quantix, Photometrics, Tucson, AZ). Imaging Workbench software was used for data acquisition and analysis. YFP (XF104-2) and CFP (XF130-2) filter cubes were purchased from Omega Optical Inc. (Brattleboro, VT).

### Glucose-6-Phosphate assay

We used a commercial G-6-P assay kit (Biovision Research Products, Mountain View, CA). CHO cells (0.5–2×10^6^) were transfected with GLUT1 (∼3 µg). Two days later cells were washed in ice cold PBS and frozen in situ in dryice ethanol. 200 µl of 6% (v/v) perchloric acid added and the cells scraped while frozen. Cells were homogenized via a “Qiashredder” column at 4°C and the homogenate neutralized with 500 mM ethanolamine and 10 M KOH [30]. Samples were then centrifuged to remove insoluble material (10 mins 15000 g 4°C) 50 µl samples were transferred into wells of a 96-well plate and brought to a volume of 100 µl with assay buffer. Absorbance at 450 nm was measured, using a POLARstar Omega microplate reader (BMG LABTECH GmbH, Offenburg/Germany). Background samples (without enzyme mix) were introduced to estimate the NADH/NADPH levels prior to G-6-P conversion. Finally, to estimate the G-6-P levels a curve was generated with G-6-P standards containing 0, 2, 4, 6, 8, 10, 20 nmol/well.

## Supporting Information

Figure S1 **Subcellular distribution of HKI linked to YFP in CHO cells.** To investigate the subcellular localization of HK in CHO cells we co-expressed HKI-CFP (A) together with the mitochondria marker MitoTracker-YFP (B). In panel C superimposition of the two images shows that the localization of HKI and MitoTracker overlap, indicating HKI association with mitochondria.
(TIF)

Figure S2 **Glucose uptake and clearance in CHO cells over expressing GLUT1 and GLUT4.** To monitor GLUT insertion into the plasma membrane, we used constructs of GLUT1 and GLUT4 linked to GFP. In both cases, there was only faint GFP fluorescence detected in the plasma membrane, with most of the fluorescence associated with intracellular membrane compartments (results not shown). However, as shown in Panels B and C, even this low level of plasma membrane GLUT insertion had dramatic effects on glucose transport. With GLUT1 over expression, the rate of glucose entry, illustrated by a decrease in FRET ratio, increased dramatically, with the time constant τ dropping from 34.5+/−5 s (n = 7) to 1.9+/−0.6 s (n = 8) (Panels 1 and 3). The rate of glucose clearance, indicated by an increase in FRET ratio also increased, with a t_1/2_ approaching 10 s (9.8+/−3.5 s). With GLUT4 over expression, the rate of glucose uptake decreased by almost 14-fold, from 34.5 s to 2.5 s (Panels 1 and 5). The rate of glucose clearance also increased in this case, although to a lesser level than with GLUT1, the time to reach half clearance (t_1/2_) being around 12.5+/−6.5 s (n = 7). In panels 1, 3 and 5 the rate of glucose uptake was fitted to an exponential decay, in panels 2, 4 and 6 glucose clearance was fitted to a sigmoidal function (see Materials and Methods for details). These data suggest that increased insertion of GLUTs in the plasma membrane increase both glucose influx and efflux.
(TIF)

Figure S3 **Effects of GLUT1 on glucose utilization in the presence of Cyto B in CHO cells.** Panel A shows data obtained with a CHO cell exposed to Cyto B (20 µM) in the continuous presence of 10 mM glucose. The right hand side plots show the rate of glucose clearance following removal of glucose in the absence of Cyto B (A1) and in the presence of Cyto B (B1). The closeness of the two τs suggests that, with cells exhibiting a slow glucose uptake phenotype, GLUT-mediated glucose efflux has little effect in glucose clearance. This trace also illustrates that the effect of Cyto B is readily reversible, as glucose entry resumed, albeit at a lower rate, 30 s after Cyto B removal. In B data were obtained with a CHO cell transfected with GLUT1, which exhibited a high rate of glucose uptake. In this case, Cyto B dramatically reduced the rate of clearance as compared to that recorded in the absence of Cyto B and after outside glucose removal (plots B3 and B4). These data suggest that GLUT-mediated efflux plays a role in glucose clearance in cells overloaded with glucose.
(TIF)

Figure S4 **siRNA-mediated depletion of HKI and HKII.** To study the role of HKs in glucose clearance we transfected CHO cells with 30 to 40 nM of HKI or HKII siRNA. (A) Inhibition of HKI expression caused a decrease in the rate of glucose clearance with the time to reach half clearance (t_1/2_) increasing from 75+/−15 s to 135+/−22 s (n = 9). (B) Similarly, inhibition of HKII expression decreased glucose clearance with t_1/2_ reaching 152+/−20 s (n = 9). These results indicate that endogenous HKI and HKII equally contribute to glucose clearance in CHO cells. The data obtained with RT-PCR corroborate this result and show that the levels of endogenous HKI and HKII are very similar in these cells.
(TIF)

Table S1 **Quantification of HKI and HKII levels in CHO cells.** We have used the RT-PCR method to estimate the levels of HKI and HKII in native cells and in cells over expressing HKI and HKII. Our results indicate that the level of these two enzymes is similar. This is consistent with our observation where depletion of endogenous HKI and HKII decrease glucose clearance to similar extend. This quantitative analysis also shows that cell culture in the absence of glucose causes a slight increase in HK levels. This is important when considering the effect of glucose removal on glucose utilization. Indeed, we have shown that incubation in the absence of glucose decreases glucose clearance and we hypothesized that this effect was related to HK translocation away from mitochondria. Our quantitative analysis supports this hypothesis and shows that the effect on glucose clearance is not due to a decrease in HK level. Finally, we show, as expected, that over expression of HKs is accompanied by an increased level of intracellular HK. Thus, the decrease in glucose clearance observed with HKII over expression may be related to increased level of the enzyme in the cytoplasm, favoring glycogen synthesis.
(DOC)
