# Supplementary figures and images for: Subcellular Localization of Hexokinases I and II Directs the Metabolic Fate of Glucose
Source: PLoS One. 2011 Mar 9;6(3):e17674. doi: 10.1371/journal.pone.0017674 (PMC3052386; doi:10.1371/journal.pone.0017674)

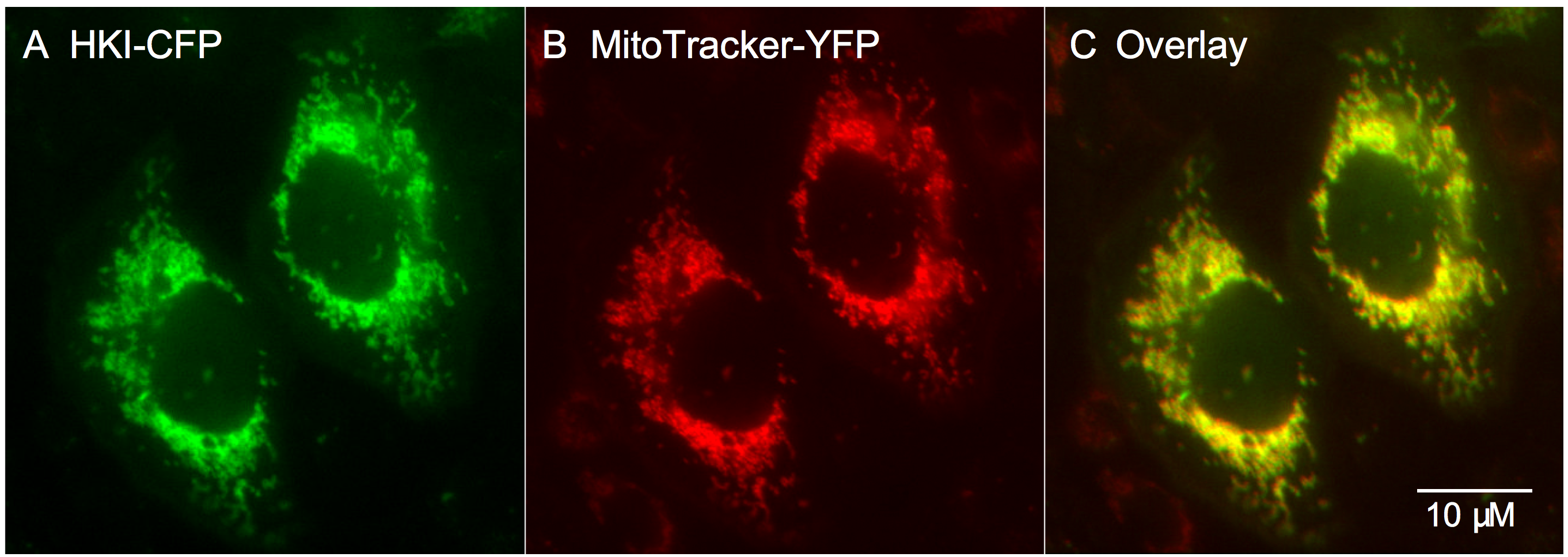

Supplement: Figure S1 — Subcellular distribution of HKI linked to YFP in CHO cells. To investigate the subcellular localization of HK in CHO cells we co-expressed HKI-CFP (A) together with the mitochondria marker MitoTracker-YFP (B). In panel C superimposition of the two images shows that the localization of HKI and MitoTracker overlap, indicating HKI association with mitochondria. (TIF) [file pone.0017674.s001.tif]

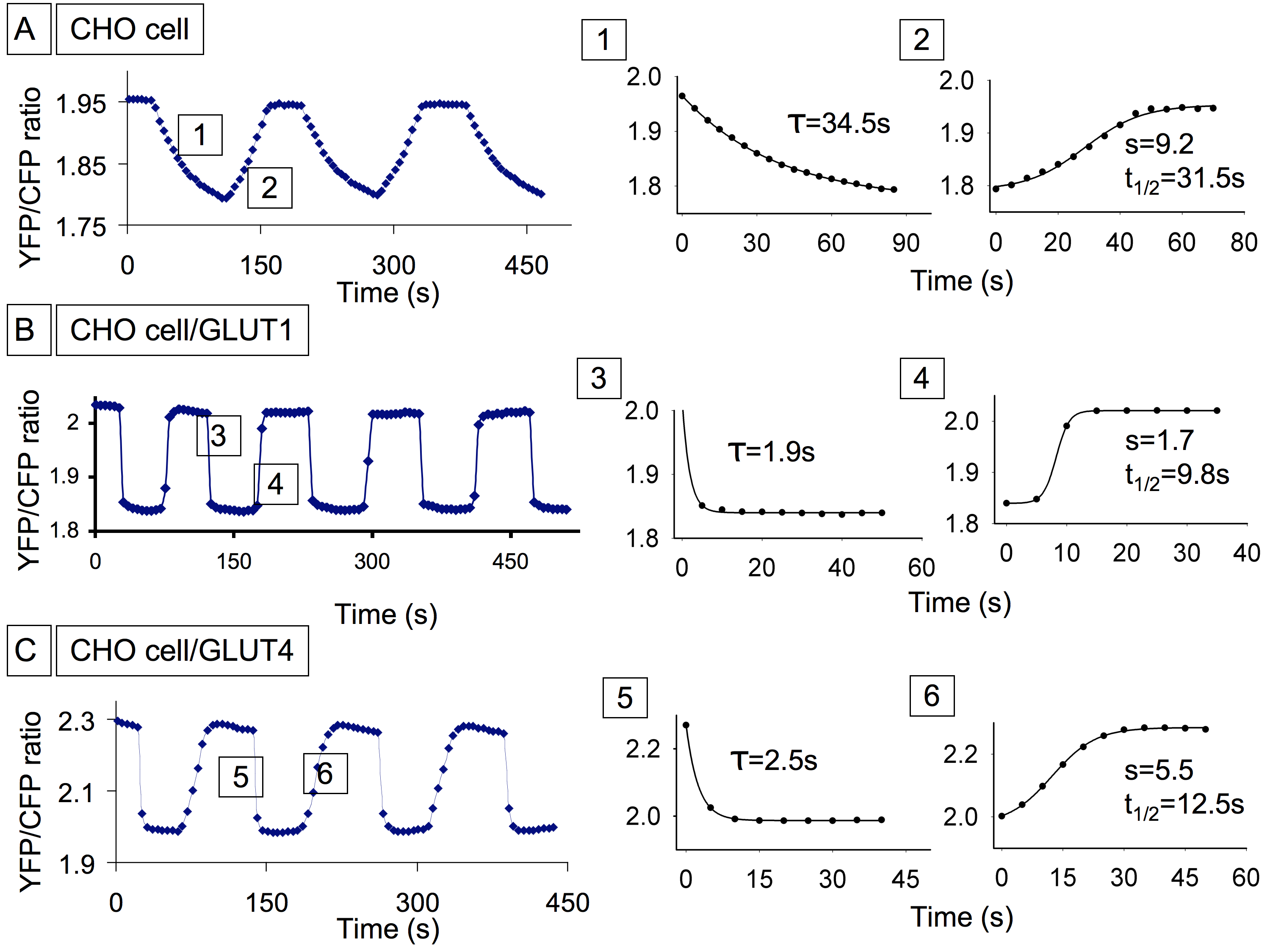

Supplement: Figure S2 — Glucose uptake and clearance in CHO cells over expressing GLUT1 and GLUT4. To monitor GLUT insertion into the plasma membrane, we used constructs of GLUT1 and GLUT4 linked to GFP. In both cases, there was only faint GFP fluorescence detected in the plasma membrane, with most of the fluorescence associated with intracellular membrane compartments (results not shown). However, as shown in Panels B and C, even this low level of plasma membrane GLUT insertion had dramatic effects on glucose transport. With GLUT1 over expression, the rate of glucose entry, illustrated by a decrease in FRET ratio, increased dramatically, with the time constant τ dropping from 34.5+/−5 s (n = 7) to 1.9+/−0.6 s (n = 8) (Panels 1 and 3). The rate of glucose clearance, indicated by an increase in FRET ratio also increased, with a t1/2 approaching 10 s (9.8+/−3.5 s). With GLUT4 over expression, the rate of glucose uptake decreased by almost 14-fold, from 34.5 s to 2.5 s (Panels 1 and 5). The rate of glucose clearance also increased in this case, although to a lesser level than with GLUT1, the time to reach half clearance (t1/2) being around 12.5+/−6.5 s (n = 7). In panels 1, 3 and 5 the rate of glucose uptake was fitted to an exponential decay, in panels 2, 4 and 6 glucose clearance was fitted to a sigmoidal function (see Materials and Methods for details). These data suggest that increased insertion of GLUTs in the plasma membrane increase both glucose influx and efflux. (TIF) [file pone.0017674.s002.tif]

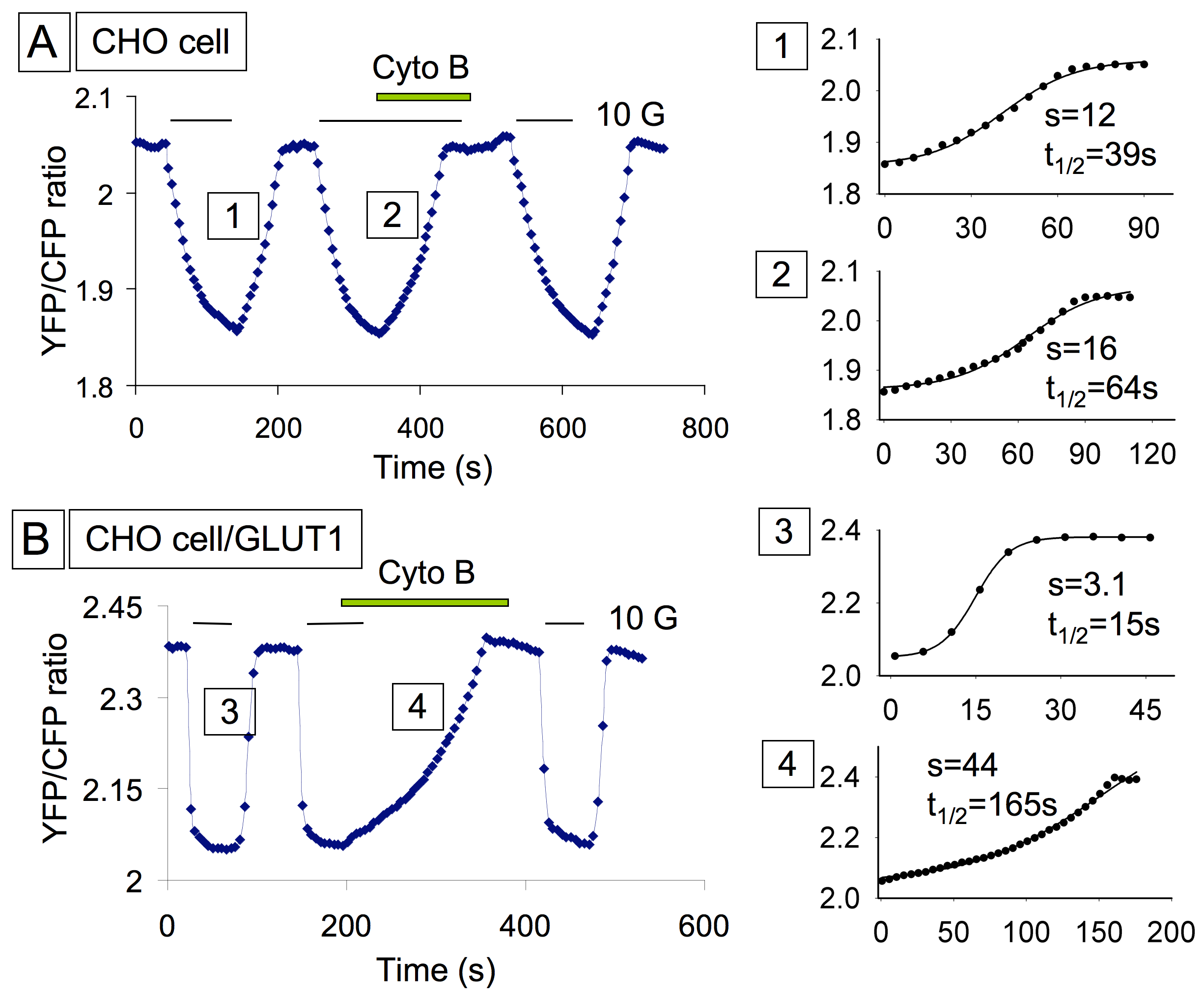

Supplement: Figure S3 — Effects of GLUT1 on glucose utilization in the presence of Cyto B in CHO cells. Panel A shows data obtained with a CHO cell exposed to Cyto B (20 µM) in the continuous presence of 10 mM glucose. The right hand side plots show the rate of glucose clearance following removal of glucose in the absence of Cyto B (A1) and in the presence of Cyto B (B1). The closeness of the two τs suggests that, with cells exhibiting a slow glucose uptake phenotype, GLUT-mediated glucose efflux has little effect in glucose clearance. This trace also illustrates that the effect of Cyto B is readily reversible, as glucose entry resumed, albeit at a lower rate, 30 s after Cyto B removal. In B data were obtained with a CHO cell transfected with GLUT1, which exhibited a high rate of glucose uptake. In this case, Cyto B dramatically reduced the rate of clearance as compared to that recorded in the absence of Cyto B and after outside glucose removal (plots B3 and B4). These data suggest that GLUT-mediated efflux plays a role in glucose clearance in cells overloaded with glucose. (TIF) [file pone.0017674.s003.tif]

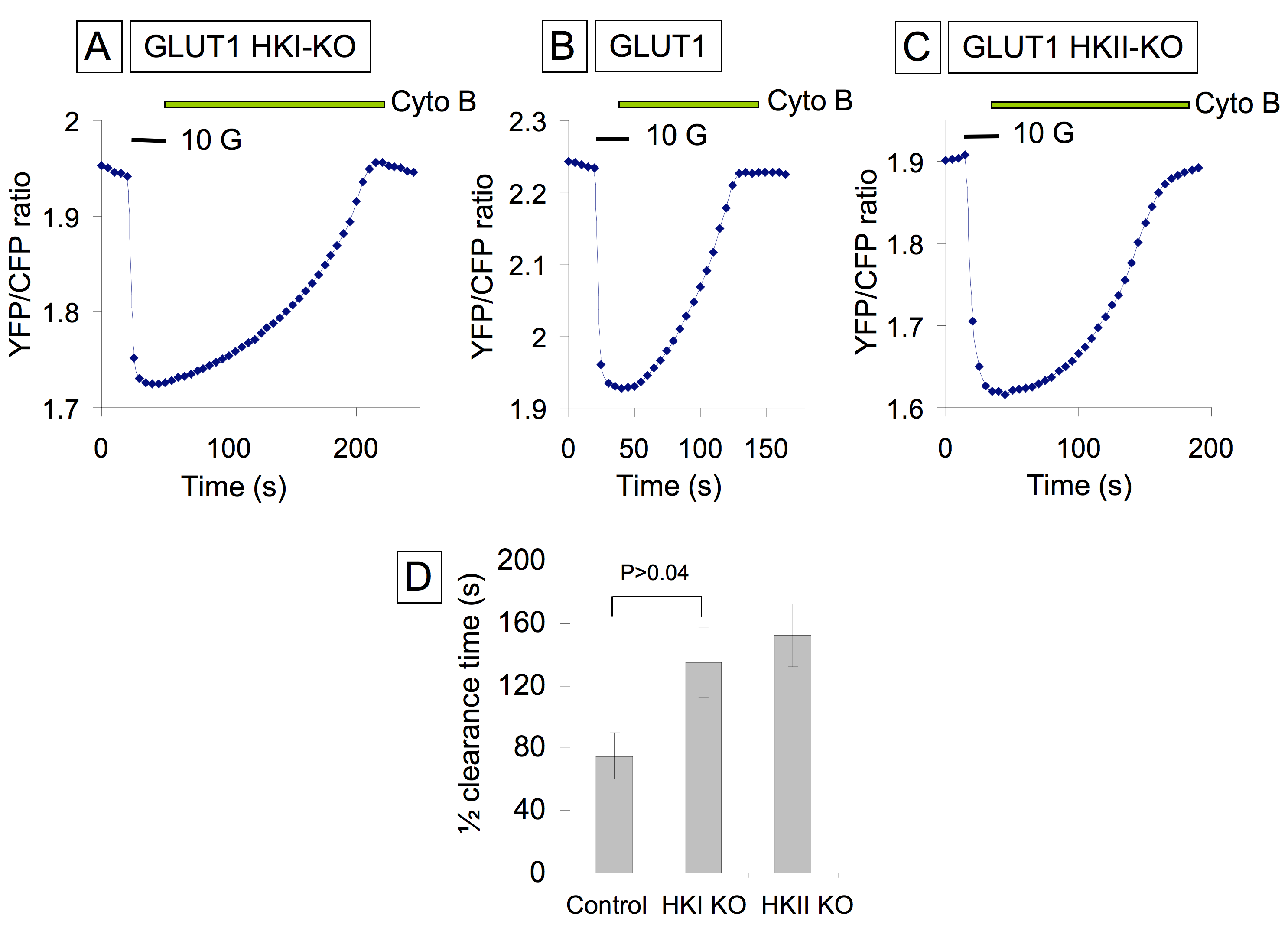

Supplement: Figure S4 — siRNA-mediated depletion of HKI and HKII. To study the role of HKs in glucose clearance we transfected CHO cells with 30 to 40 nM of HKI or HKII siRNA. (A) Inhibition of HKI expression caused a decrease in the rate of glucose clearance with the time to reach half clearance (t1/2) increasing from 75+/−15 s to 135+/−22 s (n = 9). (B) Similarly, inhibition of HKII expression decreased glucose clearance with t1/2 reaching 152+/−20 s (n = 9). These results indicate that endogenous HKI and HKII equally contribute to glucose clearance in CHO cells. The data obtained with RT-PCR corroborate this result and show that the levels of endogenous HKI and HKII are very similar in these cells. (TIF) [file pone.0017674.s004.tif]
