# Supplementary material for: Subcellular Localization of Hexokinases I and II Directs the Metabolic Fate of Glucose
Source: PLoS One. 2011 Mar 9;6(3):e17674. doi: 10.1371/journal.pone.0017674 (PMC3052386; doi:10.1371/journal.pone.0017674)
Supplement: Table S1 — Quantification of HKI and HKII levels in CHO cells. We have used the RT-PCR method to estimate the levels of HKI and HKII in native cells and in cells over expressing HKI and HKII. Our results indicate that the level of these two enzymes is similar. This is consistent with our observation where depletion of endogenous HKI and HKII decrease glucose clearance to similar extend. This quantitative analysis also shows that cell culture in the absence of glucose causes a slight increase in HK levels. This is important when considering the effect of glucose removal on glucose utilization. Indeed, we have shown that incubation in the absence of glucose decreases glucose clearance and we hypothesized that this effect was related to HK translocation away from mitochondria. Our quantitative analysis supports this hypothesis and shows that the effect on glucose clearance is not due to a decrease in HK level. Finally, we show, as expected, that over expression of HKs is accompanied by an increased level of intracellular HK. Thus, the decrease in glucose clearance observed with HKII over expression may be related to increased level of the enzyme in the cytoplasm, favoring glycogen synthesis. (DOC) [file pone.0017674.s005.doc]

|  |  | Treatment |  |
| --- | --- | --- | --- |
|  | 0 Glucose 1hr | 0 Glucose 24 hr | HKII overexpression |
| HKI fold change | 1.2 | 1.6 | -1.6 |
| HKII fold change | -1.3 | 1.2 | 28.4 |
